# Consequences of specialized breeding in the Swedish Warmblood horse population

**DOI:** 10.1111/jbg.12731

**Published:** 2022-07-13

**Authors:** Sandra Bonow, Susanne Eriksson, Emma Thorén Hellsten, Åsa Gelinder Viklund

**Affiliations:** ^1^ Department of Animal Breeding and Genetics Swedish University of Agricultural Science Uppsala Sweden; ^2^ Swedish Warmblood Association Flyinge Sweden

**Keywords:** breeding value, dressage, genetic parameters, jumping, sport horses, Young Horse Test

## Abstract

In many European warmblood studbooks, clear specialization toward either jumping or dressage horses is evident. The Swedish Warmblood (SWB) is also undergoing such specialization, creating a possible need for separate breeding programs and a discipline‐specific Young Horse Test (YHT). This study investigated how far specialization of the SWB breed has proceeded and the potential consequences. Individuals in a population of 122,054 SWB horses born between 1980 and 2020 were categorized according to pedigree as jumping (J), dressage (D), allround (AR), or thoroughbred (Th). Data on 8,713 J horses and 6,477 D horses assessed for eight traits in YHT 1999–2020 were used to estimate genetic parameters within and between J and D horses and between different periods. Future scenarios in which young horses are assessed for either jumping or dressage traits at YHT were also analyzed. More than 80% of horses born in 1980–1985 were found to be AR horses, while 92% of horses born in 2016–2020 belonged to a specialized category. The average relationship within J or D category was found to increase during the past decade, whereas the relationship between these categories decreased. Heritability estimates for gait traits were 0.42–0.56 for D horses and 0.25–0.38 for J horses. For jumping traits, heritability estimates were 0.17–0.26 for J horses and 0.10–0.18 for D horses. Genetic correlations between corresponding traits assessed in J and D horses were within the range 0.48–0.81, with a tendency to be lower in the late study period. In the future scenarios, heritability and genetic variance both decreased for traits that were not assessed in all horses, indicating that estimation of breeding value and genetic progress for these traits could be affected by a specialized YHT. However, ranking of sires based on estimated breeding values (EBVs) and accuracy of EBVs was only slightly altered for discipline‐specific traits. With continued specialization in SWB, specialization of the YHT should thus be considered.

## 1 INTRODUCTION

The Swedish Warmblood (SWB) is the most common horse breed in Sweden, with approximately 65,000 registered horses and 2,800 foals born per year (HNS, 2021), although SWB is still a small population in comparison with other European warmblood studbooks (WBFSH, 2021). The SWB has its origin in the eighteenth century, when its main purpose was to supply royal cavalry with suitable riding horses (Graaf, 2004). When the studbook was established in 1928, the aim was to breed horses for multiple equestrian purposes. During recent decades, breeding has focused more on sport performance, and the current breeding goal for SWB is to produce competitive horses at international level in show jumping and dressage (Swedish Warmblood Association, 2021). As a result, breeding has become more specialized toward either jumping or dressage as demonstrated by Ablondi et al. (2019) in a study based on high‐density single‐nucleotide polymorphism (SNP) array data, which found genetic differences between SWB horses bred for show jumping and dressage.

Specialization of breeding for sport disciplines has also occurred in other European warmblood studbooks, for example, it has been reported for the Dutch warmblood (KWPN) (Rovere et al., 2014). In the KWPN studbook, foals have been registered as either jumping or dressage horses since 2006. Another example is the Oldenburg studbook, which was divided into two specialized studbooks (for show jumping and dressage) in 2001 (Oldenburger Pferdezuchtverband, 2022). In the Danish Warmblood studbook, a breeding plan with the focus on separation of the disciplines was initiated in 2004, and young horse assessments are now discipline‐specific (Dansk Varmblod, 2022). In SWB, the genetic trend for specialization in show jumping or dressage increased considerably from the mid‐1980s, mainly because of strong stallion selection and import of high‐quality stallions for either jumping or dressage (Viklund et al., 2011). Since 2002, the SWB stallion performance test has been specialized, and stallions are approved for breeding based on results from only one discipline (Granberg, 2017). Since 2019, it is also possible to choose discipline in the Riding Horse Test for 4‐year‐olds (RHT), provided that the horse has already participated in the Young Horse Test (YHT), where all traits are assessed, as a 3‐year‐old (SWB, 2018). However, according to the breeding organization, some owners of dressage horses would like to be able to opt out of having jumping assessed (Thorén Hellsten, 2022). Similarly, owners of jumping horses often do not pay much attention to assessments of walk and trot.

The ongoing specialization of SWB creates a possible need for separate breeding programs, making it necessary to investigate the consequences that this can have for the rather small SWB population. If some horses are not assessed for all traits in YHT, this could affect the estimated genetic variance and heritability as well as estimation of breeding values for these traits. In the long term, a potentially lower heritability and accuracy of breeding values could have a negative impact on genetic improvement of the breed.

The aim of the present study was to investigate how far the specialization process of SWB toward subpopulations of jumping and dressage horses has proceeded, and the consequences this could have for selection strategy and genetic evaluation in this relatively small population. To fulfil this aim, genetic parameters were estimated, relationships within and between SWB subpopulations were compared, and future scenarios in which horse owners could choose to have their young horses assessed for only one discipline in YHT were investigated.

## 2 MATERIALS AND METHODS

### 2.1 Data

A pedigree file for 315,117 was provided by the Swedish Warmblood Association. In addition, breeding values (EBVs) for show jumping and dressage were obtained from the routine genetic evaluation in 2020. Information about the horse participation in YHT and RHT as well as in official competition was also provided. The study population was restricted to SWB horses born between 1980 and 2020, where SWB horses were defined as horses with a SWB ID number and no foreign number in the database. Furthermore, only horses sired by a stallion with a SWB studbook number or at least 10 assessed offspring at YHT or RHT were included. In total, 122,054 horses met these criteria. The number of sires was 1,581, and the number of grandsires was 1,315, with 1,157 stallions appearing as both sires and grandsires.

### 2.2 Classification

Sires and grandsires were assigned to one of four categories: jumping (J), dressage (D), allround (AR), or thoroughbred (Th). The classification was performed by the breeding director for SWB. Sires approved in stallion performance test in or after 2002 were easily assigned to a category because they were assessed for only one discipline at the test (jumping or dressage). Sires approved before 2002 were assigned to the J or the D category according to pedigree, breeding values, own performance, and offspring performance. Sires with verified good performance in both jumping and dressage or sires who had offspring which had demonstrated good performance in both jumping and dressage were assigned to the AR category. These sires could have either jumping or dressage as their main discipline. Sires used in breeding in the beginning of the study period often had no obvious specialization in either jumping or dressage and were therefore classified as AR. The Th category consisted of English Thoroughbred (xx) and Arabian Thoroughbred (ox). Anglo‐Arabian Thoroughbred (x) sires were not classified in the Th category because these horses are mainly bred for riding, whereas the other Thoroughbred breeds are mainly bred for racing. The Anglo‐Arabian Thoroughbred sires used in SWB breeding had intermediate breeding values in both jumping and dressage and was therefore classified as AR.

The horses in the population were classified according to the sire's category, except if they had a category J sire and a category D grandsire, or vice versa, in which case they were classified as AR. A majority of the horses were classified as AR (46,262), followed by J (41,279) and D (29,822). Fewer horses were classified as Th (4,691).

### 2.3 Traits

The provided EBVs from the 2020 routine genetic evaluation had been estimated in two separate multi‐trait models for jumping and dressage including data from competition, YHT, and RHT according to methods described in Viklund et al. (2011). Lifetime accumulated points in show jumping or dressage competition, transformed with a logarithm to the basis of 10, are the breeding goal traits, while evaluating scores from YHT and RHT are early indicator traits that have been shown to be strongly correlated to the breeding goal traits (Viklund et al., 2011). Horses that are placed, that is, are among the 25% best in each competition receive points. A horse receives more points either for a better placing or at a more advanced level or both. EBVs for the breeding goal traits show jumping and dressage from the routine genetic evaluation are considered in this study.

In YHT, eight traits are subjectively assessed by two judges using a scale from 1 to 10, where 10 is the best score (Table 1). One judge assesses conformation, walk, and trot and one judge assesses jumping traits, while canter is assessed jointly by both judges. YHT results for 19,621 horses evaluated between 1999 (when YHT was introduced) and 2020 and a pedigree file with seven generations from tested horses were used in further analysis. The data from the YHT were divided into two 11‐year periods named “early” (1999–2009) and “late” (2010–2020), referring to horses born 3 years before that, that is, 1996–2006 and 2007–2017. The traits in the full dataset and in the different time periods were treated as different traits to enable comparisons. Competition data included official results from competitions in show jumping, dressage, and eventing at the regional, national, and international level.

**TABLE 1:** Mean,^1^ standard deviation (SD), minimum (Min) and maximum (Max) value of traits in Young Horse Test, assessed for jumping horses (*N* = 8713) and dressage horses (*N* = 6477)

Trait	Jumping horses				Dressage horses			
	Mean	SD	Min	Max	Mean	SD	Min	Max
Type	7.71^a^	0.62	4.0	10.0	7.87^b^	0.64	4.0	10.0
Head‐neck‐body	7.53^a^	0.55	4.0	9.0	7.70^b^	0.57	5.0	9.5
Correctness of legs	7.32^a^	0.62	4.0	9.0	7.32^a^	0.65	2.0	9.0
Walk at hand	7.04^a^	0.68	4.0	10.0	7.52^b^	0.75	4.0	10.0
Trot at hand	6.72^a^	0.68	4.0	9.5	7.47^b^	0.84	4.0	10.0
Free canter	7.28^a^	0.75	4.0	10.0	7.35^b^	0.80	3.0	10.0
Free jumping—TA^2^	7.41^a^	1.14	1.0	10.0	6.13^b^	1.09	1.0	10.0
Free jumping—TG^3^	7.32^a^	1.29	1.0	10.0	6.25^b^	1.18	1.0	10.0

^1^ Mean values between jumping and dressage horses with different superscripts were significantly different (*p* < .05).

^2^ Technique and ability.

^3^ Temperament and general impression.

### 2.4 Future scenarios

To illustrate a possible future situation where horse owners can choose to have their horse assessed for only one discipline in YHT, two alternative scenarios were created and compared with the current situation. In the first scenario, it was assumed that approximately 50% of D horses (those with the lowest scores for jumping) were not assessed for jumping traits. In the second scenario, it was assumed that approximately 50% of J horses (those with the lowest scores for walk and trot) were not assessed for these traits. The limit for removing observations was set as the categorical value closest to the median of each group, which resulted in the following data edits:

*D spec*: For D horses, scores for the two jumping traits “Technique and ability” (TA) and “Temperament and general impression” (TG) were removed from the dataset if the sum of the two scores was lower than 12.5 points. This eliminated 52% of the D horses.

*J spec*: For J horses, scores for walk and trot were removed from the dataset if the sum of the two scores was lower than 14 points. This eliminated 42% of the J horses.

Two ranking lists, on the basis of EBVs, were created for sires with at least 10 tested offspring at YHT. The first, named “J sire group”, consisted of 217 J sires and AR sires with jumping as their main discipline. The second, named “D sire group”, consisted of 182 D sires and AR sires with dressage as their main discipline. Spearman's rank correlations were estimated to quantify re‐ranking according to the scenarios compared with the current situation. This was performed separately for the J and D sire groups.

### 2.5 Genetic analysis

Descriptive statistics on the data were analyzed using SAS (Statistical Analysis System) (SAS Institute Inc., 2015). The average relationships between and within categories were computed using the software package CFC (Sargolzaei et al., 2006), using an indirect approach as described by Colleau (2002). Trends for relationships were estimated by computing the relationship for each category in eight birth‐year periods of 5 years between 1980 and 2020 (with the exception of the first group, which covered 6 years). Genetic parameters and EBVs for traits assessed at YHT were estimated using the DMU program package, version 6 (Madsen & Jensen, 2013). Traits from YHT were analyzed similarly to the routine genetic evaluation (Viklund et al., 2011), with the following animal model:

$$Y_{\mathrm{ijk}}=\mu +{\text{event}}_{i}+{\mathrm{sex}}_{j}+a_{k}+e_{\mathrm{ijk}}$$

where *Y*_*ijk*_ is the observed value of horse *k*; *μ* is the population mean; event_*i*_ is the fixed effect of location‐date combination, *i* = 1,2…547; sex_*j*_ is the fixed effect of sex, *j* = male or female; *a*_*k*_ is the additive genetic effect of horse *k* ~ ND (0, $A\sigma_{a}^{2}$) (where A is the relationship matrix; $\sigma_{a}^{2}$ is the additive genetic variance), and *e*_*ijk*_ is the random ~IND (0, $\sigma_{e}^{2}$) residual effect (where $\sigma_{e}^{2}$ is the residual variance).

Genetic parameters and EBVs were estimated for all YHT traits using univariate analysis. Genetic correlations between corresponding traits assessed for category J and category D horses were estimated using bivariate analysis. Because each horse was assigned to one category, J or D, the residual covariance was then set to 0. Bivariate analyses were also used to estimate the genetic correlations between jumping and gait traits for all horses. Accuracy of EBVs was calculated for the J and D sire groups and for the two scenarios. Accuracy was defined as the correlation between true and estimated breeding value (*r*_TI_) and was calculated as

$$r_{\mathrm{TI}}=\sqrt{1-\mathrm{PEV}/\sigma_{a}^{2}},$$

where PEV is prediction error variance, calculated as in (Henderson, 1975).

## 3 RESULTS

### 3.1 Population structure

Of the 1,581 sires included in the analysis, 713 were classified as J, 487 were classified as D, and 288 were classified as AR. The sires in category AR consisted of 141 horses classified as “jumping‐dressage”, a further 141 classified as “dressage‐jumping”, and six horses of Anglo‐Arabian breed (x). There were 93 sires in category Th (84 English Thoroughbreds and nine Arabian Thoroughbreds).

**FIGURE 1:**
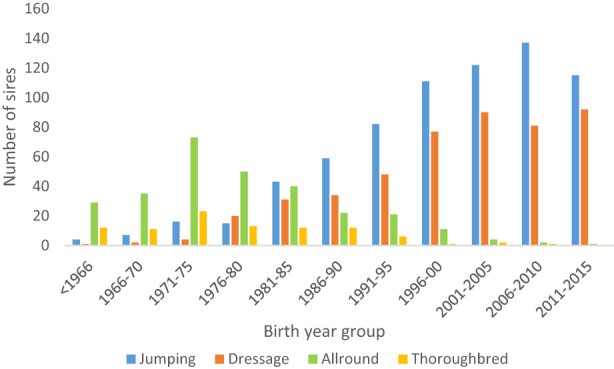
Number of sires in Swedish Warmblood breeding by category and birth year group [Colour figure can be viewed at wileyonlinelibrary.com]

**FIGURE 2:**
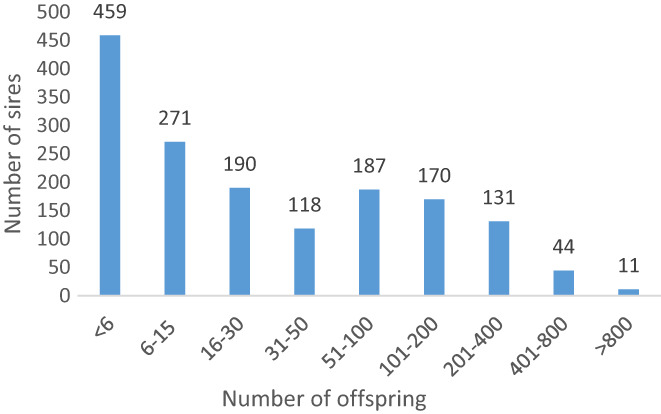
Distribution of sires approved for SWB breeding according to number of offspring [Colour figure can be viewed at wileyonlinelibrary.com]

The majority of the sires born before 1980 were classified as AR (59%) (Figure 1). From the early 1980s, more sires came to be bred for either jumping or dressage, and the number of AR sires started to decline. The proportion of sires classified as AR born 1971–1975 were 63%, and 30 years later, (2001–2005) the number had decreased to 1.8%. In the latest birth year group (2011–2015), a majority of the sires were classified as J (55%), followed by D (45%), while the number of AR sires was close to 0 (Figure 1). The number of Th sires also decreased to become almost nonexistent in 2020. The total number of available sires increased considerably in recent decades, and the number of offspring per sire decreased. Of the 1,581 sires analyzed, a majority had 30 or fewer offspring (Figure 2), with a total range from 1 to 1,386 offspring per sire.

**FIGURE 3:**
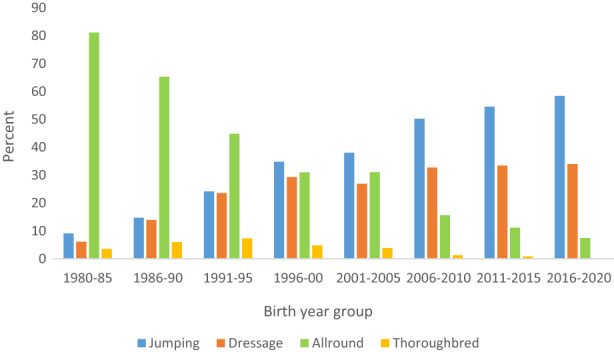
Distribution (%) of horses in the Swedish Warmblood population into different categories according to birth year group between 1980 and 2020 [Colour figure can be viewed at wileyonlinelibrary.com]

A majority of the 122,054 horses investigated were classified as AR (37.9%), followed by J (33.8%) and D (24.4%). A small number of horses were classified as Th (3.8%). Figure 3 shows changes in the distribution of the categories according to birth year. In the beginning of the study period, AR horses were in the great majority. By 2020, about 58% of the population consisted of J horses, 34% of D horses, and 7.5% of AR horses. Horses in category Th, that is, horses with a Thoroughbred sire were relatively common in the 1990s but declined to close to zero by 2020 (Figure 3).

**FIGURE 4:**
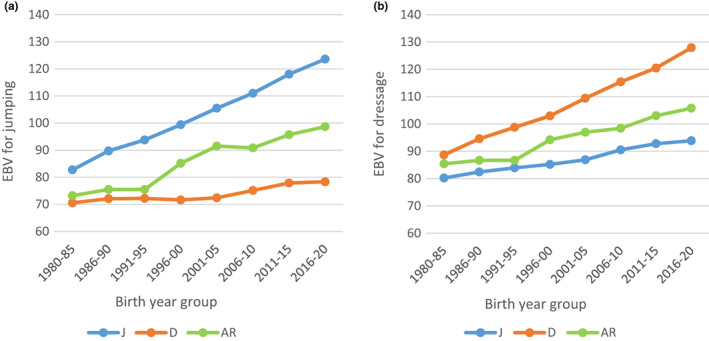
Estimated breeding values (EBV) for (a) show jumping and (b) dressage according to birth year group for Swedish Warmblood horses classified as jumping (J), dressage (D), or allround (AR) horses [Colour figure can be viewed at wileyonlinelibrary.com]

For J horses, the average EBV from the routine genetic evaluation for jumping increased from 83 to 124 between 1980 and 2020, whereas the increase in average EBV for jumping was very modest for D horses (from 71 to 78) (Figure 4a). On the other hand, the average EBV for dressage increased from 89 to 128 for D horses, while the increase in average EBV for dressage for J horses was smaller, from 80 to 94 (Figure 4b). For AR horses, the increase in average EBV was intermediate (relative to the changes for D and J horses) for both disciplines.

### 3.2 Relationship

**FIGURE 5:**
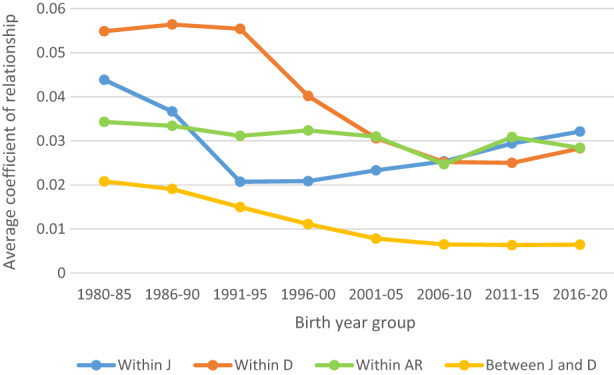
Average coefficient of relationship as a function of birth year group within and between categories of Swedish Warmblood horses classified as jumping (J), dressage (D), or allround (AR) [Colour figure can be viewed at wileyonlinelibrary.com]

At the beginning of the study period, D horses had a higher average relationship coefficient within the category (5.5%) compared with J horses (4.4%) (Figure 5). The average relationship among J horses decreased considerably during the 1980s. There was also a drastic decrease for D horses, but starting about 10 years later. By 2020, the average relationship was increasing within both these categories, while the average relationship coefficient between the categories had declined to a very low level (<1%). In contrast, the level of relationship within the category of AR horses remained around 3% from 1980 until 2020.

### 3.3 Performance

**FIGURE 6:**
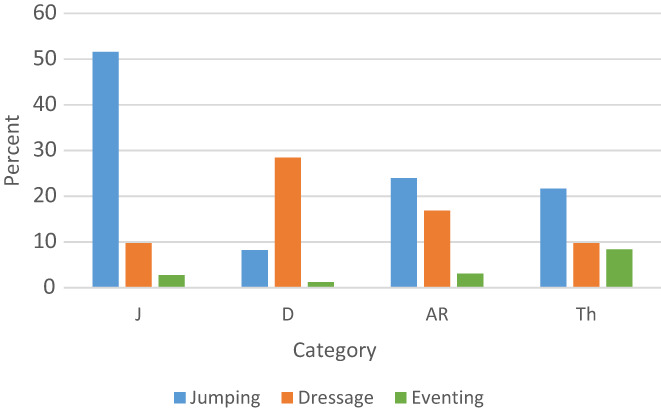
Percentage of horses born 1980–2016 competing in show jumping, dressage, or eventing, stratified by their category: Jumping (J), dressage (D), allround (AR), or thoroughbred (Th) [Colour figure can be viewed at wileyonlinelibrary.com]

About 30% of the population born 1980–2017 was assessed in either YHT or RHT, or both. Around 40% of the population competed, with most horses competing in jumping (29%), followed by dressage (17%). Only a small proportion (2.8%) of the population competed in eventing. About 50% of J horses competed in jumping, whereas only 28% of D horses competed in dressage (Figure 6). For AR horses, more competed in jumping (24%) compared with dressage (17%). Category Th had the highest proportion of horses competing in eventing (8.4%) (Figure 6).

Category D horses born in 1996–2017 and assessed at YHT had considerably higher mean values for walk and trot compared with J horses (Table 1). On the other hand, J horses had substantially higher mean values for the two jumping traits (TA and TG). The mean values for type, head–neck‐body, and canter were slightly higher for D horses than J horses, whereas the mean value for correctness of legs was similar for both categories. The differences between J and D horses for all mean values of corresponding traits except correctness of legs were statistically significant (*p* < 0.05).

### 3.4 Genetic parameters

For the 8,713 J horses assessed in YHT, the heritability of traits was low to moderate (range 0.17–0.38) (Table 2). For the 6,477 D horses, the heritability was moderate to high for conformation and gaits (0.30–0.56) and low for the two jumping traits (0.10–0.18). For all horses in total (19,621), the heritability was moderate to high for all traits (0.23–0.53), except for correctness of legs (0.08). For all horses in total, the genetic variance varied between 0.03 and 0.16 for conformation traits and between 0.18 and 0.41 for gaits and jumping traits. For J and D horses, the genetic variance ranged from 0.07 to 0.36 and was somewhat higher for D horses for all traits, except for the two jumping traits. In comparison with all horses in total, D horses had similar or higher heritability and genetic variance for all traits except jumping traits, while J horses had lower heritability and genetic variance for all traits (Table 2). The genetic correlations between gait traits and jumping traits for all horses were close to zero or low and unfavorable for walk and trot (from 0 to −0.13) and moderate and positive for canter (0.25–0.30). Standard errors for the correlations were between 0.04 and 0.05.

**TABLE 2:** Estimated heritability (*h* ^2^) with standard error as subscript, genetic ($\sigma_{a}^{2}$ ) and residual ($\sigma_{e}^{2}$) variances for traits evaluated in young horse test for horses classified as jumping or dressage and for all horses in total

Trait	Jumping horses			Dressage horses			All horses		
	*h* ^2^	$\sigma_{a}^{2}$	$\sigma_{e}^{2}$	*h* ^2^	$\sigma_{a}^{2}$	$\sigma_{e}^{2}$	*h* ^2^	$\sigma_{a}^{2}$	$\sigma_{e}^{2}$
Type	0.32_0.03_	0.12	0.26	0.48_0.04_	0.19	0.21	0.40_0.02_	0.16	0.24
Head–neck‐body	0.23_0.03_	0.07	0.22	0.30_0.04_	0.10	0.22	0.30_0.02_	0.09	0.22
Corr. of legs	^a^	^a^	^a^	^a^	^a^	^a^	0.08_0.01_	0.03	0.35
Walk at hand	0.25_0.03_	0.11	0.33	0.42_0.04_	0.23	0.31	0.36_0.02_	0.18	0.32
Trot at hand	0.38_0.03_	0.17	0.28	0.56_0.04_	0.36	0.28	0.53_0.02_	0.29	0.26
Free canter	0.33_0.03_	0.17	0.36	0.45_0.04_	0.27	0.33	0.35_0.02_	0.20	0.37
Free jumping – TA	0.26_0.03_	0.33	0.92	0.18_0.03_	0.20	0.94	0.31_0.02_	0.41	0.90
Free jumping – TG	0.17_0.02_	0.27	1.33	0.10_0.02_	0.13	1.21	0.23_0.02_	0.36	1.23

*Note*: The horses were born in 1996–2017.

Abbreviations: TA, technique and ability; TG, temperament and general impression.

^a^ Did not reach convergence.

The heritability and genetic variance for YHT traits were estimated for both the early period (1999–2009) and the late period (2010–2020) and compared with the corresponding parameters for all horses in total (1999–2020) (Tables 3 and 4). For J horses, the heritability was lower for head–neck‐body and free canter in the late period, whereas the heritability for the other traits was similar for both periods. For D horses, the heritability was higher for type, head–neck‐body, and free canter in the late period, whereas the heritability for the other traits remained unchanged over time (Table 3).

**TABLE 3:** Estimated heritability (with standard error as subscript) in Young Horse Test (YHT) for horses classified as jumping or dressage horses in the early period (1999–2009), late period (2010–2020) and total period (1999–2020) and for all horses in the total period (1999–2020)

Trait	Jumping horses			Dressage horses			All horses
	Early	Late	Total	Early	Late	Total	Total
Type	0.32_0.05_	0.30_0.04_	0.32_0.03_	0.41_0.06_	0.49_0.06_	0.48_0.04_	0.40_0.02_
Head‐neck‐body	0.31_0.05_	0.15_0.03_	0.23_0.03_	0.19_0.05_	0.31_0.05_	0.30_0.04_	0.30_0.02_
Corr. of legs	^a^	^a^	^a^	^a^	^a^	^a^	0.08_0.01_
Walk at hand	0.19_0.04_	0.22_0.04_	0.25_0.03_	0.46_0.06_	0.43_0.05_	0.42_0.04_	0.36_0.02_
Trot at hand	^a^	0.42_0.05_	0.38_0.03_	^a^	0.64_0.05_	0.56_0.04_	0.53_0.02_
Free canter	0.43_0.05_	0.25_0.04_	0.33_0.03_	0.42_0.06_	0.51_0.06_	0.45_0.04_	0.35_0.02_
Free jumping—TA^b^	0.28_0.05_	0.24_0.04_	0.26_0.03_	0.15_0.04_	0.13_0.04_	0.18_0.03_	0.31_0.02_
Free jumping—TG^c^	0.18_0.04_	0.18_0.03_	0.17_0.02_	0.08_0.03_	0.06_0.03_	0.10_0.02_	0.23_0.02_

^a^ Did not reach convergence.

^b^ Technique and ability.

^c^ Temperament and general impression.

**TABLE 4:** Estimated genetic variance (with standard error as subscript) for traits in Young Horse Test for horses classified as jumping or dressage in the early period (1999–2009), late period (2010–2020) and total period (1999–2020) and for all horses in the total period (1999–2020)

Trait	Jumping horses			Dressage horses			All horses
	Early	Late	Total	Early	Late	Total	Total
Type	0.13_0.02_	0.11_0.02_	0.12_0.01_	0.19_0.03_	0.17_0.02_	0.19_0.02_	0.16_0.01_
Head‐neck‐body	0.10_0.02_	0.04_0.01_	0.07_0.01_	0.06_0.02_	0.09_0.02_	0.10_0.01_	0.09_0.01_
Corr. of legs	^a^	^a^	^a^	^a^	^a^	^a^	0.03_<0.01_
Walk at hand	0.09_0.02_	0.09_0.02_	0.11_0.01_	0.28_0.04_	0.21_0.03_	0.23_0.02_	0.18_0.01_
Trot at hand	^a^	0.17_0.02_	0.17_0.02_	^a^	0.38_0.04_	0.36_0.03_	0.29_0.01_
Free canter	0.28_0.04_	0.11_0.02_	0.17_0.02_	0.28_0.05_	0.27_0.04_	0.27_0.03_	0.20_0.01_
Free jumping—TA^b^	0.42_0.07_	0.25_0.04_	0.33_0.04_	0.19_0.06_	0.12_0.04_	0.20_0.04_	0.41_0.03_
Free jumping—TG^c^	0.34_0.07_	0.25_0.05_	0.27_0.04_	0.13_0.05_	0.07_0.03_	0.13_0.03_	0.36_0.03_

^a^ Did not reach convergence.

^b^ Technique and ability.

^c^ Temperament and general impression.

In the late period, the genetic variance for J horses was lower for all traits except walk at hand (Table 4). For D horses, the genetic variance was lower in the late period or unchanged. The heritability and genetic variance were in general higher for D horses than J horses, except for the two jumping traits.

The genetic correlations between traits at YHT evaluated in J and D horses in the early period, late period, and in total are presented in Table 5. The genetic correlation ranged between 0.48 and 0.81 for the total period but varied between the early and late period, and the values were associated with high standard error and were not significantly different from unity. The results indicated lower genetic correlations between J horses and D horses in the late period than in the early period for all traits except free canter, but none of these correlations were significantly different.

**TABLE 5:** Estimated genetic correlation (with standard error as subscript) for traits in Young Horse Test between horses classified as jumping or dressage in the early period (1999–2009), late period (2010–2020) and total period (1999–2020)

Trait	Genetic correlation		
	Early	Late	Total
Type	0.97_0.20_	0.74_0.22_	0.75_0.14_
Head‐neck‐body	0.89_0.29_	0.65_0.39_	0.78_0.18_
Corr. of legs	^a^	^a^	^a^
Walk at hand	0.55_0.30_	−0.11_0.41_	0.48_0.20_
Trot at hand	^a^	0.70_0.19_	0.81_0.12_
Free canter	0.64_0.22_	0.82_0.25_	0.73_0.15_
Free jumping—TA^b^	0.82_0.38_	0.52_0.46_	0.72_0.23_
Free jumping—TG^c^	0.56_0.56_	0.22_0.77_	0.56_0.33_

^a^ Did not reach convergence.

^b^ Technique and ability.

^c^ Temperament and general impression.

### 3.5 Future scenarios

The heritability and genetic variance for traits assessed at YHT and the corresponding parameters for scenario *D spec* and scenario *J spec* are presented in Table 6. When the results of the poorest performing horses were excluded, heritability and genetic variance both decreased. For the jumping traits, also the residual variance decreased. The sire rank correlations between EBVs from analysis of *Full data* (with all results in YHT included) and scenario *D spec* or scenario *J spec* are presented separately for the J and D sire groups in Table 7. For the J sire group, the correlation between *Full data* and scenario *D spec* was very high for jumping traits, while the correlation between *Full data* and scenario *J spec* was moderate for walk and trot. For the D sire group, the opposite was seen, with a very high correlation between *Full data* and scenario *J spec* (walk and trot) and low to moderate correlation between *Full data* and scenario *D spec* (jumping traits). When no results were removed, the ranking was the same, with a correlation equal to 1.00.

**TABLE 6:** Estimated heritability (h^2^) and genetic (σ^2^_a_) and residual (σ^2^_e_) variance (with standard error as subscript) for traits in Young Horse Test, based on *Full data* ^a^ and in future scenarios *D spec* and *J spec* ^b^

Trait	Full data			Scenario D spec			Scenario J spec		
	*h* ^2^	$\sigma_{a}^{2}$	$\sigma_{e}^{2}$	*h* ^2^	$\sigma_{a}^{2}$	$\sigma_{e}^{2}$	*h* ^2^	$\sigma_{a}^{2}$	$\sigma_{e}^{2}$
Walk at hand	0.36_0.02_	0.18_0.01_	0.32_0.01_	–	–	–	0.30_0.02_	0.13_0.01_	0.30_0.01_
Trot at hand	0.53_0.02_	0.29_0.01_	0.26_0.01_	–	–	–	0.48_0.03_	0.23_0.01_	0.25_0.01_
Free jumping—TA^c^	0.31_0.02_	0.41_0.03_	0.90_0.02_	0.24_0.02_	0.25_0.03_	0.79_0.02_	–	–	–
Free jumping—TG^d^	0.23_0.02_	0.36_0.03_	1.23_0.02_	0.15_0.02_	0.20_0.03_	1.12_0.03_	–	–	–

^a^ Full data: including all results for horses evaluated 1999–2020.

^b^ D‐spec: for D horses with the poorest results for TA and TG, scores for these traits were omitted in the analyses. J‐spec: for J horses with the poorest results for walk and trot at hand, scores for these traits were omitted in the analyses.

^c^ Technique and ability.

^d^ Temperament and general impression.

**TABLE 7:** Correlations between rankings for *full data* ^a^ and future scenarios *D spec* and *J spec* ^b^ *,* shown separately for the jumping sire and dressage sire groups

Trait	Jumping sire group		Dressage sire group	
	Full data ‐ D spec	Full data ‐ J spec	Full data ‐ D spec	Full data ‐ J spec
Walk at hand	1.00	0.56	1.00	0.96
Trot at hand	1.00	0.60	1.00	0.97
Free jumping – TA	0.95	1.00	0.48	1.00
Free jumping – TG	0.93	1.00	0.19	1.00

Abbreviations: TA, technique and ability; TG, temperament and general impression.

^a^ Full data: Including all results from horses evaluated 1999–2020.

^b^ D‐spec: for D horses with the poorest results for TA and TG, score for these traits were omitted in the analyses. J‐spec: for J horses with the poorest results for walk and trot at hand, score for these traits were omitted in the analyses.

The accuracy of EBVs for *Full data* and for scenarios *D spec* and *J spec* is presented separately for the J and D sire group in Table 8. For both scenarios evaluated, the mean accuracy value (r_TI_) decreased in comparison with when all results were included (*Full data*). The largest differences were seen for jumping traits in the D sire group (18%–25% decrease), while the decrease for walk and trot in the J sire group was smaller (11%–14%).

**TABLE 8:** Mean accuracy (r_TI_) for breeding values (with standard deviation as subscript) for *Full data* ^a^ and for future scenarios *D spec* and *J spec* ^b^ and difference in accuracy (Diff.) between *Full data* and each scenario, shown separately for the jumping sire and dressage sire groups

Trait	Jumping sire group				Dressage sire group			
	Full data	D spec	J spec	Diff. (%)	Full data	D spec	J spec	Diff. (%)
Walk at hand	0.87_0.06_	–	0.75_0.13_	−13.8	0.86_0.08_	–	0.80_0.12_	−7.0
Trot at hand	0.91_0.05_	–	0.81_0.11_	−11.0	0.90_0.07_	–	0.85_0.11_	−5.6
Free jumping—TA^c^	0.85_0.07_	0.78_0.12_	–	−8.2	0.84_0.09_	0.69_0.13_	–	−17.9
Free jumping—TG^d^	0.82_0.08_	0.72_0.13_	–	−12.2	0.81_0.10_	0.61_0.14_	–	−24.7

^a^ Full data: including all results from horses evaluated 1999–2020.

^b^ D‐spec: for D horses with the poorest results for TA and TG, score for these traits were omitted in the analyses. J‐spec: for J horses with the poorest results for walk and trot at hand, score for these traits were omitted in the analyses.

^c^ Technique and ability.

^d^ Temperament and general impression.

## 4 DISCUSSION

### 4.1 Classification

In this study, horses in the SWB population were divided into categories according to the category of their sire and grandsire. The classification of sires was in some cases performed subjectively, which could be regarded as a weakness of the study. However, the results showed clear and logical differences between J and D horses, indicating that the classification was in general correctly performed. In contrast to the study by Rovere et al. (2014), horses in our study were classified not only as J or D but also as AR or Th. This probably made the classification in our study more refined. Additionally, in the present study, J and D horses were classified according to their sire's discipline, meaning that a sire could only have offspring in one of the two categories. In the study by Rovere et al. (2014), 40% of the sires had offspring in both the J and D subpopulations.

### 4.2 Discipline specialization

A clear specialization over time toward either J or D horses was evident, with the number of AR sires (Figure 1) and AR horses (Figure 3) declining considerably during the last decade of the study period in favor of J and D horses. Based on the trends in EBVs (Figure 4), there were large differences between J and D horses in terms of average EBVs for jumping and dressage. Similarly, mean values in YHT for walk and trot on one hand and jumping traits on the other showed clear differences between J and D horses (Table 1).

In a previous study, Viklund et al. (2011) investigated genetic trends in SWB for show jumping and dressage and found a considerable increase in EBVs for both disciplines, starting in the mid‐1980s. They also found that the 50% best sires in each discipline had noticeably better EBVs in comparison with the mean of all sires. A similar finding was made for the 50% best dams in comparison with the mean of all dams. This indicates that the specialization into disciplines of the SWB breed is a process that has been going on for at least 40 years.

### 4.3 Relationship

A decrease in relationship between J and D horses and an increase in relationship within the groups of J and D horses were seen in this study (Figure 6). Even so, the average relationship within category was still low (approximately 3% for both categories). Similarly, Rovere et al. (2014) found that genetic connections between the two subpopulations “jumping” and “dressage” in KWPN decreased markedly after the studbook was divided in 2006, while the relationship within the subpopulations increased. The average relationship coefficient they found for KWPN was slightly higher than that in our study (4% for J horses and 5% for D horses, for horses born in 2009). Rovere et al. (2014) concluded that if the specialization process continues, it will give rise to two unrelated subpopulations in KWPN. This would probably also be seen in SWB if separation into different breeding programs was to be introduced.

Our result is a further confirmation of what is reported in Ablondi et al. (2019), where they found genetic differences between SWB horses bred for show jumping or dressage by analyzing high‐density SNP array data. The horses in that study were born in 2010–2011, and the authors found signatures of selection in 11 chromosomes. The selected regions included genes with known function in mentality, endogenous reward system, development of connective tissues and muscles, motor control, body growth, and development.

In the beginning of our study period (1980s), the relationship within the groups of J and D horses was higher than in 2020. This could be partly explained by the low number of horses classified as J or D at that time. In the 1980s, there was a drastic decrease in relationship for J horses, and 10 years later, the same was seen for D horses, probably due to importation of stallions with bloodlines that had not been used previously in the SWB breed. Today, those bloodlines are well spread in all European warmblood studbooks (Ruhlmann et al., 2009), which could partly explain the increase in relationship seen for both categories in recent years. In a future scenario with separate breeding programs, the average increase in relationship needs to be monitored carefully to avoid inbreeding.

### 4.4 Heritability estimates

The moderate to high heritability estimates, except for correctness of legs (Table 2), in analysis of the full dataset were in agreement with findings by Viklund and Eriksson (2018). In the study by Rovere et al. (2017), the heritability for conformation and gait traits was slightly lower (0.24–0.39) than in the present study (0.35–0.53). The heritability for trot showed the highest value in both studies. The heritability for jumping found by Rovere et al. (2017) (0.33) was slightly higher than in this study (range 0.23–0.31).

Category D horses had higher heritability for gaits than J horses, whereas the opposite was seen for jumping traits. The genetic variance for these traits showed a similar pattern. This may indicate use of a wider assessment scale for gaits in D horses than in J horses and for jumping traits in J horses than in D horses. Use of a wider assessment scale in these cases depends primarily on more frequent use of higher scores for discipline‐specific traits within category. In the study by Rovere et al. (2015), the differences between heritability estimates for J and D horses were smaller, for example, for conformation and gait, heritability ranged from 0.26 to 0.37 for D horses and from 0.21 to 0.39 for J horses.

Compared with all horses, D horses had similar or higher heritability and genetic variance for all traits except jumping traits. This may indicate that division into subpopulations would be favorable for D horses since high heritability and genetic variance are advantageous for genetic progress. On the other hand, the heritability and genetic variance for J horses were lower for all traits, in comparison with all horses, so division into subpopulations might have negative impacts on genetic progress for J horses.

On comparing genetic parameters estimated for the early (1999–2009) and late (2010–2020) periods (Table 3), no clear pattern could be seen. Similarly, Rovere et al. (2015) found no clear trend between different periods (1998–2002, 2003–2007, and 2008–2012) for conformation and gait traits in the KWPN population. In the study of SWB by Viklund et al. (2010b), higher heritability and genetic variance estimates were found in the later period (1988–2007) compared with the earlier period (1973–1987) for horses assessed in RHT. Those authors concluded that the higher heritability in the later period could have been be due to improvements in judging of horses and that the increase in genetic variance could have been due to import of sires and increased specialization into either jumping and dressage. However, the periods studied by Viklund et al. (2010b) were longer than in the present study and thus covered many changes in both sport and breeding, which may explain why those authors found differences between periods that were not detected in this study.

### 4.5 Genetic correlations

The genetic correlations between corresponding traits for J and D horses were weaker in the late compared with the early period for all traits except free canter (Table 5). The results indicated that there were larger differences between J and D horses for traits in the late period, which could confirm an ongoing specialization process in the population. However, the standard error was very high in most cases, making it difficult to draw any firm conclusions. Similar results were seen in the study by Rovere et al. (2015), in which the genetic correlations between gait traits assessed for J and D horses in the later period (2008–2012) were lower than those in the earlier period (2003–2007) (jumping traits were not included in that study).

In contrast to other traits, the genetic correlation for free canter between J and D horses was higher in the late period. This could possibly be explained by the cooperation between the two judges when assessing this trait. During the assessment, the judge assessing gaits and the judge assessing jumping discuss and jointly set a score for canter, which may increase the correlation between J and D horses for this trait.

The estimated genetic correlations between gait traits and jumping traits were higher than corresponding estimates in a study by Viklund et al. (2008), where data from YHT between 1999 to 2003 were investigated. Viklund et al. (2008) concluded that walk and trot appeared to have no genetic relationship with jumping traits (−0.05 to 0.03), while the correlations they observed between canter and jumping traits were stronger (0.32–0.33). The weak correlations between disciplines in the SWB population were confirmed in a later study in which Viklund et al. (2010a) found slightly negative to low genetic correlations between jumping traits judged in RHT and dressage competition (−0.19 to 0.17) and walk and trot judged under rider in RHT and show jumping (−0.01 to 0.18). In the KWPN population, Rovere et al. (2017) observed a negative correlation between jumping traits assessed at studbook entry inspection and dressage competition (−0.39), while the assessments of walk and trot were not correlated with show jumping. Their estimated genetic correlation between show jumping and dressage was weak and unfavorable (−0.21), and they concluded that a breeding program under specialization might be most effective if separate breeding goals were defined for each discipline. In the SWB population, there is a need for further investigation of the correlations between YHT results and competition data in order to identify characteristics that are important for successful sport horses in both disciplines.

### 4.6 Future scenarios

Two different scenarios were assessed, in which scores for walk and trot and jumping traits were omitted for approximately 50% of the poorest performing J and D horses, respectively. This could illustrate a situation where the horse owner is aware that their horse would probably receive at most a modest score for jumping (or walk and trot) and thus chooses to have their horse assessed as either a jumping horse or a dressage horse. Scenario *D spec*, in which 50% of D horses were not assessed in jumping, is a likely future scenario because some owners of D horses appear to find free jumping unnecessary. There is also a higher risk of a horse that is not fully trained for free jumping having a bad experience in the test, which would be undesirable from an animal welfare perspective.

Scenario *J spec*, in which 50% of J horses were not assessed for walk and trot, could also represent a future scenario because owners of J horses are often less interested in assessment of gaits other than canter. However, walk and trot are less time‐consuming for judges to assess, which could be a reason for retaining these traits in the assessment. This is also the practice in KWPN (Rovere et al., 2015), where all horses are assessed for gaits regardless of discipline. Another argument for assessing all traits at YHT is to encourage horse owners to give their horse a varied training, which is considered to have positive impacts on health and durability (Braam et al., 2011).

In both scenarios, the estimated heritability and genetic variance declined for walk, trot, and jumping traits in comparison with the current situation (Table 6). Lower heritability could slow down genetic progress in the population, which could be an argument for retaining assessments for all traits. However, while the genetic variance appeared to be reduced in the scenarios, the consequences may be less severe in practice. If the genetic variance for a trait is large mainly because of weak performance by horses of a different discipline, genetic progress will probably not decline if these horses are not assessed since they were not intended for that discipline in any case. This assumes that the ranking of selected candidates is not affected.

Our analysis of future scenarios indicated that the ranking of sires on EBVs for jumping traits would only be slightly affected for the J sire group in scenario *D spec*, that is, when some of the D horses were not assessed in free jumping (Table 7). A similar pattern was seen for walk and trot for the D sire group in scenario *J spec*, that is, when some J horses were not assessed for these traits. This could be a reason for allowing horse owners to choose to have their horse assessed as either a jumping horse or a dressage horse since it is more important to have an accurate evaluation for jumping traits for J sires and for walk and trot for D sires. On the other hand, the re‐ranking was strongly affected for the opposite traits, that is, evaluation of jumping traits for the D sire group and evaluation of walk and trot for the J sire group. The accuracy of EBVs for these traits also decreased to a larger extent in both scenarios, in comparison with the full dataset (where all results were included) (Table 8). This indicates that genetic evaluation for these traits would be affected and give less certain EBVs if some horses were not assessed for all traits at YHT. This could make it more difficult for breeders aiming to produce horses with high quality in both gaits and jumping traits, such as AR horses and eventers.

## 5 CONCLUSIONS

This analysis revealed clear specialization into jumping or dressage horses in the SWB breed. The average relationship within these categories increased in the past decade, while the relationship between horses in different categories decreased. Evaluation of two different future scenarios, in which horse owners could choose to have their young horses assessed as either a jumping horse or a dressage horse in YHT, showed that both estimated heritability and genetic variance decreased for traits that were not assessed in all horses. This could have a negative impact on genetic progress for these traits. The ranking of sires (based on EBVs) was also altered in both scenarios, which could have consequences for estimation of breeding values. However, since there were only minor changes for traits connected to the respective disciplines, that is, jumping traits for J horses and walk and trot for D horses, specialization of the YHT can be a viable option, provided that the number of horses participating in YHT stays at the same level and that interest in producing AR horses remains low.

## Data Availability

The data that support the findings of this study are available from Swedish Warmblood Association. Restrictions apply to the availability of these data, which were used under license for this study. Data are available from the author(s) with the permission of Swedish Warmblood Association.
